# The cost of community research—recruiting community-dwelling participants to a feasibility primary care cluster randomised controlled trial

**DOI:** 10.1186/s13063-021-05297-3

**Published:** 2021-05-08

**Authors:** Nicola Harrison, Caroline Brundle, Anne Heaven, Andrew Clegg

**Affiliations:** 1 Bradford Institute for Health Research, Bradford Teaching Hospitals NHS Foundation Trust, Bradford, UK; 2 Academic Unit of Ageing and Stroke Research, University of Leeds, Leeds, UK

**Keywords:** Consent, Recruitment, Community-dwelling, Time, Cost

## Abstract

**Background:**

To support a robust evidence base for the organisation and provision of community-delivered health services for older people, clinical trials need to be designed to account for community-based participant recruitment. There is currently little reported information available on the time and cost of recruiting community-dwelling older people, which makes the completion of cost attribution documentation problematic when applying for research funding.

**Main body:**

We aimed to establish the amount of researcher time it takes to recruit community-dwelling older people to a feasibility primary care cluster randomised controlled trial, including collecting baseline data. The trial was part of a programme of work investigating an intervention to improve the quality of life for older people with frailty. Two researchers conducting home visits to recruit and collect baseline data from participants recorded the time spent on travelling to and from the visit, at the visit itself and any associated administration. The median total researcher activity time per visit was 148 min. We discuss the various elements of recruitment and data collection activity and the factors that impacted the length of time taken, including location, individuals’ capacity and cognition, hearing and visual impairment and the desire for social contact.

**Conclusion:**

Studies cannot reach their recruitment targets if they are unrealistically planned and resourced. We recommend that trials recruiting older people in the community allocate two and a half hours of researcher time per person, on average, for consent, baseline data collection, travel and administration. We acknowledge that a variety of different factors will mean that researcher activity will vary between different community-based trials. Our findings give a good starting point for timing calculations, and evidence on which to base the justification of research activity costings.

**Trial registration:**

Personalised care planning for older people with frailty ISRCTN12363970. 08/11/2018.

## Background

A key policy focus for the 2019 NHS Long Term Plan is for health services to be provided to people in their communities [1]. Older people are likely to be target recipients of many new community-delivered health services. To support a robust evidence base for the organisation and delivery of such services, clinical trials need to be designed to account for community-based participant recruitment.

Reviews of published, publicly funded randomised controlled trials (RCTs) in the UK have found that only around half reached their target sample size [2, 3] and nearly half required an extension [3]. There is a considerable body of literature on different techniques and strategies to use to optimise the recruitment of older people to healthcare research [4, 5]. However, a Cochrane review of methods used to increase recruitment to trials identified very few that were supported by high-quality evidence [6].

Guidance on which methods might be most effective in helping to meet recruitment targets is undoubtedly useful. However, for the purposes of planning timescales, resource use and staffing levels, more information is required. Studies have shown that *expected* and *actual* recruitment times may considerably differ [7]—possibly because these expectations have no empirical basis.

It is acknowledged that adequate time for screening, recruitment and data collection should be factored into planning research with older people [8, 9]. However, there is currently little reported time and cost information available. Recruitment and data collection are complex processes, but data from studies recruiting participants from the community, including older people, lacks detail, only providing a broad overview of the timings for either recruitment and screening [10] or data collection [9].

The development of research grant applications and the completion of cost attribution documentation, such as the Schedule of Events Cost Attribution Template (SoECAT) [11]—which is now required for health and care studies funded by the National Institute for Health Research (NIHR) in the UK—are problematic without accurate information on the timings for different aspects of research activity.

## The PROSPER case study

We aimed to establish the amount of researcher time it takes to recruit community-dwelling older people to the Personalised Care Planning for Older People with Frailty (PROSPER) feasibility primary care cluster randomised controlled trial [12]. This included collecting baseline data from recruited participants.

The PROSPER Programme aims to establish whether personalised care planning (PCP) can improve quality of life for older people with frailty and reduce overall health and social care costs. A total of 343 participants were recruited into the feasibility trial between March 2019 and January 2020. Eligible individuals were sent study information by general practices (GPs), and those expressing interest in taking part in the trial were visited at home in order to gain consent and undertake baseline data collection.

Two researchers conducting home visits, within an approximate 13-mile radius of the research base, timed all elements of every third visit undertaken in September and October 2019. This timing period represented approximately 20% of the total recruitment time, during which participants were being recruited from 12 different general practices. These months were chosen as they were outside of school holidays and seasonal extreme weather and would therefore involve ‘typical’ travel conditions.

The time spent on travelling to and from the visit by car (travel time), time spent in face-to-face participant contact at the home visit itself (participant contact time) and any associated administration time were recorded on a bespoke pro-forma.

Participant contact time was further broken down into eight categories covering the following:

- Discussing the study (including an assessment of capacity to consent)
- Gaining informed consent
- Undertaking screening with the Montreal Cognitive Assessment (MoCA) (to establish cognitive ability to engage with the intervention)
- Baseline data collection (the 20-page baseline assessment case report form (CRF) included six measures of health and quality of life along with questions about healthcare and social care use [12])
- Consultee declaration (in cases where it was deemed there was a lack of capacity to consent)
- Gaining carer consent
- Baseline carer data collection
- Miscellaneous time (any time taken up during the home visit with activity not related to the research)

Not all categories were relevant to every visit, e.g. those related to consultees or carers.

Administration time was broken down into completing paperwork including obtaining information from GP records, correspondence and registering patients onto research databases.

The median, interquartile range (IQR) and range were calculated for each activity category and for total researcher activity time—the sum of the individual components.

## Results

### Timing taken on visits

Data were collected from 33 visits, and 29 of these individuals were recruited onto the trial; the remaining four declined participation. The median (IQR) total researcher activity time per visit was 148 (129.5–165.25) min. Total activity, however, ranged from just over 1 to just over 4 h. Table 1 shows the full breakdown of activities and times. Consultee and carer recruitment activities are not included in Table 1, due to low numbers during the timing period.

**Table 1:** Full breakdown of activities and times

	Median (IQR)	Range
**Travel time (min)**	40 (35–55)	10–100
**Participant contact time (min)**	81 (66–94)	15–126
Discuss study	20 (15–22)	10–45
Gain consent	5 (3.75–5.25)	2–10
Cognitive screening (MoCA)	13 (11–15)	9–20
Baseline data collection	37 (30–43)	19–63
Miscellaneous other	5 (5–10.5)	3–18
**Administration time (min)**	24 (22–28)	15–36
Completing paperwork (inc. obtaining GP record data)	16 (15–18)	10–20
Registering on research databases	4 (3–4)	3–10
Correspondence	6 (2–6)	2–6
**Total researcher activity time (min)**	**148 (129.5**–**165.25)**	**65**–**242**

Total participant contact lasted a median (IQR) of 81 (66–94) min, but ranged from 15 min to just over 2 h. The 15-min visit time was recorded when the individual decided not to take part following discussion with the researcher. The visit that lasted over 2 h included gaining consultee declaration (as the participant was deemed to lack capacity to give informed consent) and recruitment of a carer requiring a separate carer consent form and baseline carer questionnaire.

### Influences on activity time

*Travel time* varied considerably. The median (IQR) travel time was 40 (35–55) min. The longest round trip took 100 min, and the shortest journey time was 10 min.

Across the full recruitment period of the trial, carers were recruited for 20% of study participants; however, the number of carers recruited during the timing period was unusually low (8%). Consultee involvement was 3% across the whole recruitment period, including during timing. The *involvement of consultees* and carers added to the length of home visits. Carer activity lasted a median of 10 min, and completing the single consultee declaration during the timing period took 11 min in contrast to the median of 5 min for gaining participant consent.

Times for completing the baseline data collection ranged considerably from 19 to 63 min, with a median (IQR) of 37 (30–43) min. Some participants were able to complete the questions themselves with no support, but others required *support from the researchers*, for a number of reasons such as hearing, visual, manual and cognitive impairments. Providing support including reading out the questions, using large print answer prompts and filling out the documentation was generally more time-consuming than self-completion.

Miscellaneous time was recorded for all visits and lasted a median (IQR) of 5 (5–10.5) min but ranged from 3 to 18 min. This time covered a variety of non-research activity, such as *interruptions by third parties* (co-habitants, phone calls, visitors etc.), making drinks and *time spent chatting* about topics other than the research study.

A median (IQR) of 24 (22–28) min of administrative tasks followed each home visit, with a range of 15 to 36 min.

## Conclusions

Research studies cannot reach their recruitment targets if they are unrealistically planned and resourced. We recommend that trials recruiting older people in the community allocate two and a half hours of researcher time per person, on average, for consent, baseline data collection, travel and administration. While the majority of our home visits proceeded as planned and lasted roughly the expected duration, some visits did take longer. This cannot always be predicted and is hard to plan for. However, it should at least be acknowledged in the planning of community-based trials, and accommodated within targets and timescales where possible.

We acknowledge that a variety of different factors will mean that researcher activity will vary between different community-based trials. Our findings give a good starting point for timing calculations to be based upon.

Travel was one of the major factors impacting our total recruitment time and varied considerably even though our recruitment area was limited to two relatively small conurbations in West Yorkshire. Studies recruiting in rural settings or with large catchment areas would have to allocate more time for travelling to and from home visits.

Our baseline data collection was from a clinical trial that included six commonly used assessment measures, alongside demographic information and health and social care resource use data. Although this is likely to be reasonably representative of community-based clinical trials involving older people, baseline data collection depends on the number and type of measures that are included in a trial’s CRF. Careful preparation and role playing early in the planning of the study, ideally with volunteers who are representative of the study population, should give an indication of how long this element of home visits will take.

When planning studies with older people, special consideration should be given to factors that impact on time, such as cognition, hearing and visual impairment and, in particular, the desire for social contact. Davies et al. [9] reported that the older people participating in the Newcastle 85+ Study often made comments such as ‘I look forward to your visit’ and ‘I enjoy the company’. Although only a median of 5 min per visit, we recorded miscellaneous time for every visit. This reflects, in part, the social component of conducting research in older people’s homes. Community-based researchers have to incorporate a certain, appropriate, amount of non-research time into visits, out of respect for the individuals inviting us into their homes. This additional time should not be overlooked in project planning.

Involving personal consultees for participants lacking the capacity to consent is important in research with older people, but can be time-consuming and requires careful planning [4]. A recent review found only a small proportion of clinical trials included adults lacking the capacity to consent, even in trials involving populations characterised by impaired decision-making capacity [13]. The proportion of participants requiring consultees will depend on the target population for a trial, but any research involving older people in the community is likely to encounter a lack of capacity in the sample. These people should be included wherever possible, to reduce the risk of an unrepresentative study population, and additional time for this should be factored in to study planning.

Securing personal consultee involvement made the recruitment process more time-consuming. This often required multiple visits, extra phone calls and arranging visits to fit consultees’ schedules. It is not always clear that an individual lacks capacity when an initial telephone contact is made to arrange a home visit. So, while it may not be possible for individual researchers to anticipate which visits will become extended or repeated, it should be possible for those planning and scheduling trial recruitment periods to incorporate extra time to account for this.

In areas like Bradford, many of the first-generation migrants who settled in the 1950s and 1960s and are now in later life will not be proficient in written and spoken English. Using bilingual researchers who understand the cultural norms means that Black, Asian and Minority Ethnic (BAME) participants can be included in studies, but the process is more time-consuming than with English speakers. This is especially true when dealing with languages or dialects that have no written form, when all written information has to be translated and discussed [14]. The presence at home visits of family members intending to support and/or interpret for the participant, while welcome if that provides reassurance for the participant, can also be time-consuming. Trials recruiting from diverse older populations should be planned accordingly, allowing extra time for translation.

Recruitment is not complete when the researcher leaves the participant’s home; we recorded nearly half an hour of administrative tasks for each participant following the home visit. Many of these tasks were routine but essential, for example, accurate recording of accruals to ensure project funding was appropriately released. Administration time should therefore not be overlooked.

Although visits where the person is not recruited do not involve data collection, they still entail travel and can often lengthier discussions prior to their decision about participation. We found that removing the four visits not resulting in recruitment from our median total activity time calculation had only a small effect, suggesting that the amount of time needed is similar for people who do and do not agree to participate, though we acknowledge the small numbers involved here.

Our findings are important for anyone preparing project protocols, developing funding applications and completing SoECAT forms or similar cost attribution documents for trials involving older people, particularly those with frailty. The results are also likely to be useful for researchers planning trials for wider populations with multimorbidity, who may have similar challenges to the general older population, including physical limitations, sensory impairment and cognitive impairment. An understanding of how long the elements of community-based research with older people take is vital in informing the costs associated with trials. We recommend that similar work undertaken by colleagues recruiting from other settings, such as hospital wards and care homes, and those recruiting vulnerable groups in the community, for example, people with mental health conditions or learning disabilities, would generate useful knowledge for those planning new clinical trials in these important areas.

## Data Availability

The datasets analysed during the current study are available from the corresponding author on reasonable request.

## Abbreviations

BAME: Black, Asian and Minority Ethnic
CRF: Case report form
GPs: General practices
IQR: Interquartile range
MoCA: Montreal Cognitive Assessment
NIHR: National Institute for Health Research
PCP: Personalised care planning
PROSPER: Personalised Care Planning for Older People with Frailty
RCT: Randomised controlled trial
SoECAT: Schedule of Events Cost Attribution Template
